# Changes in Allele Frequencies When Different Genomic Coancestry Matrices Are Used for Maintaining Genetic Diversity

**DOI:** 10.3390/genes12050673

**Published:** 2021-04-29

**Authors:** Elisabeth Morales-González, Jesús Fernández, Ricardo Pong-Wong, Miguel Ángel Toro, Beatriz Villanueva

**Affiliations:** 1 Departamento de Mejora Genética Animal, INIA, Ctra. de La Coruña, km 7.5, 28040 Madrid, Spain; jmj@inia.es (J.F.); villanueva.beatriz@inia.es (B.V.); 2 Genetics and Genomics, The Roslin Institute and R(D)SVS of the University of Edinburgh, Midlothian EH25 9RG, Roslin, UK; ricardo.pong-wong@roslin.ed.ac.uk; 3 Departamento de Producción Agraria, Universidad Politécnica de Madrid, 28040 Madrid, Spain; miguel.toro@upm.es

**Keywords:** genetic diversity, allele frequencies, genomic coancestry matrix, optimal contributions

## Abstract

A main objective in conservation programs is to maintain genetic variability. This can be achieved using the Optimal Contributions (OC) method that optimizes the contributions of candidates to the next generation by minimizing the global coancestry. However, it has been argued that maintaining allele frequencies is also important. Different genomic coancestry matrices can be used on OC and the choice of the matrix will have an impact not only on the genetic variability maintained, but also on the change in allele frequencies. The objective of this study was to evaluate, through stochastic simulations, the genetic variability maintained and the trajectory of allele frequencies when using two different genomic coancestry matrices in OC to minimize the loss of diversity: (i) the matrix based on deviations of the observed number of alleles shared between two individuals from the expected numbers under Hardy–Weinberg equilibrium (**θ**_LH_); and (ii) the matrix based on VanRaden’s genomic relationship matrix (**θ**_VR_). The results indicate that the use of **θ**_LH_ resulted in a higher genetic variability than the use of **θ**_VR_. However, the use of **θ**_VR_ maintained allele frequencies closer to those in the base population than the use of **θ**_LH_.

## 1. Introduction

Genetic diversity is a prerequisite for populations to be able to face future environmental changes and to ensure long-term survival [1]. Thus, a common objective in genetic conservation programs is to minimize the loss of genetic variability. This can be achieved using the Optimal Contributions (OC) method that optimizes the contributions of candidates to the next generation by minimizing the global coancestry [2,3,4]. It has been demonstrated that OC maximizes genetic diversity measured as expected heterozygosity [5], which is proportional to the additive genetic variance of quantitative traits [6]. Controlling the loss of genetic diversity also keeps the inbreeding rate under control and therefore the risk of inbreeding depression.

A different objective in genetic conservation programs can be to maintain allele frequencies to preserve the uniqueness of a particular population, since current frequencies are the result not only of genetic drift, but also of previous selection processes [7,8,9]. Selection and drift can lead to a given allele responsible for a desirable trait at a high frequency. Moreover, trying to move the frequency to intermediate values to increase genetic variability would remove the uniqueness of the population. Thus, changes in the genetic composition of populations may be undesirable, particularly when dealing with ex situ conservation programs where the final aim is the reintroduction to the wild [9].

When the OC method is applied using pedigree information to compute coancestries, both objectives (maximum heterozygosity and maintenance of allele frequencies) are achieved [9], but this is not the case when coancestries are computed from molecular marker data. Previous studies have shown that using a coancestry matrix (**θ**) computed from large numbers of single nucleotide polymorphisms (SNPs) in OC is more efficient for maintaining diversity than using the pedigree-based coancestry matrix [10,11,12]. However, given that the highest expected heterozygosity is obtained at intermediate allele frequencies, a consequence of applying OC using a **θ** based on SNP genotypes is that the genetic composition of the population is modified [9,10,11,13,14].

Different genomic coancestry matrices have been proposed for being used in OC [10,11,15,16,17]. They include the matrix that describes deviations of the observed numbers of alleles shared by two individuals from the expected numbers under Hardy–Weinberg equilibrium [18], and those obtained from genomic relationship matrices currently used in genomic predictions [19,20]. In a recent study, Morales-González et al. [16] have shown that the expected heterozygosity retained through OC was higher when using the matrix proposed by Li and Horvitz [18] than when using different genomic relationship matrices (i.e., the VanRaden’s matrices based on Method 1 and 2 [19] and the Yang’s matrix [20]). However, as mentioned above, the genomic **θ** used in OC will have an impact not only on the diversity maintained, but also on the trajectory of the change in allele frequencies. Gómez-Romano et al. [21] suggested that while OC using a genomic coancestry matrix that simply measures the proportion of alleles shared by two individuals [22] and that correlates perfectly with Li and Horvitz’s matrix favors solutions that tend to move allele frequencies towards 0.5, OC using VanRaden’s matrices would lead to solutions that tend to keep allele frequencies closer to those in the original population (i.e., allele frequencies would tend to be unchanged). This has been recently confirmed by Meuwissen et al. [17] in the context of OC aimed at maximizing genetic gain through selection while restricting the increase in inbreeding (i.e., restricting the loss of genetic diversity).

In general, populations under conservation programs are small and genetic drift leads to a loss of diversity and changes in allele frequencies. The magnitude of these drift effects depends on the effective population size (*N_e_*) which can be estimated from genomic coancestry. However, Toro et al. [23] have recently questioned the meaning of *N_e_* when genomic matrices are used in OC. In particular, when optimal management is carried out using marker information, genetic diversity can increase in the initial generations implying negative estimates of *N_e_*. Moreover, in the long term, *N_e_* does not attain an asymptotic value, but it shows an unpredictable behavior. Their findings were based on OC using Nejati-Javaremi´s matrix [22] and it is unclear if they hold when other genomic coancestry matrices are used.

The objective of this study was to evaluate, through computer simulations, the genetic variability maintained and the trajectory of allele frequencies when different genomic coancestry matrices are used in OC. Estimates of *N_e_* obtained from the change in heterozygosity computed from different genomic matrices were also compared.

## 2. Materials and Methods

Scenarios simulated involved the management of populations through the OC method using two different genomic coancestry matrices, for 50 discrete generations. Management started from a base population with family structure. The same base population was used for the 100 replicates run and it was created in two steps. Firstly, a population at mutation-drift equilibrium was generated. Secondly, the population was expanded in order to have enough individuals for sampling the 100 replicates (see below, in Section 2.1). The simulations were carried out with our own Fortran 90 codes.

### 2.1. Generation of the Base Population

The simulation of the base population was done in two steps to simulate a realistic amount of linkage disequilibrium and to ensure independency among the replicates. The first step was to generate a population in LD using a mutation-drift equilibrium approach. For this, 10,000 discrete generations of random mating for a population of 100 individuals (50 males and 50 females) were simulated. Using a larger population size would have generated an unrealistically low LD. Sires and dams were sampled with replacement and were mated at random. Each mating produced 2 offspring (1 of each sex). Thus, *N_e_* was equal to 100. The genome was composed of 20 chromosomes of 1 Morgan each. Two types of biallelic loci (SNP and unobserved loci) were simulated and they differed simply in their subsequent use. SNP loci were used for computing the genomic coancestry matrices used in the management of the population that started after the base population was created. The unobserved loci were used for measuring diversity and changes of allele frequencies, and for estimating *N_e_* across generations. Thus, the effect of different management strategies (i.e., using different genomic coancestry matrices) can be evaluated in the rest of the genome and not only on the loci used in the management (i.e., it is sometimes done using SNPs). A total of 500,000 SNPs and 500,000 unobserved loci were simulated per chromosome. At the initial generation, all loci were fixed. The mutation rate per locus and generation (μ) was 2.5 × 10^−6^ for all loci. The number of new mutations per generation was sampled from a Poisson distribution with mean 2*N_e_n_c_μn_l_*,, where *n_c_* is the number of chromosomes (i.e., 20) and *n_l_* is the total number of loci per chromosome (i.e., 1,000,000). Mutations were then randomly distributed across individuals, chromosomes and loci, switching allele 1 to allele 2 and vice versa. When generating the gametes, the number of crossovers per chromosome was drawn from a Poisson distribution with mean equal to 1. Crossovers were randomly distributed without interference. At the end of the process, the expected heterozygosity measured at both types of loci had stabilized (mutation-drift equilibrium). The second step consisted of expanding this population so we could sample the individuals to be used at the first generation of each replicate. The population was expanded during 4 generations with the aim of having enough individuals to sample 100 different replicates. During the 4 generations of expansion, each individual was randomly allocated to 8 different mates and each mating produced 1 offspring. In this way, the number of individuals in the population was multiplied by 4 each generation. After these 4 generations, the population was composed by 25,600 individuals and constituted the base population (*t* = 0). There were a total of 56,017 SNPs and 55,840 unobserved loci still segregating in *t* = 0. The expected heterozygosity (*H_e_*) computed with all loci (SNPs and unobserved loci) still segregating was 0.1811 and the linkage disequilibrium (measured as *r^2^*, the squared correlation between pairs of loci) between consecutive loci was 0.131.

### 2.2. Management Strategies

Management was performed on populations of two different sizes (*N* = 20 and *N* = 100 individuals, half of each sex) using the OC method across 50 generations. Population size was kept constant across generations. The founder individuals for each replicate were randomly sampled from the base population. Note that, given that the set of individuals sampled in *t* = 0 differs across replicates, the number of segregating loci can also differ. In most scenarios (see below, at the end of this section), all loci segregating in *t* = 0 were used for managing the population, for measuring diversity and changes of allele frequencies, and for estimating *N_e_*.

The problem to be solved in the OC method is related to the allocation of contributions, i.e., the number of offspring each candidate should produce the next generation. The pursued strategy is to minimize the global coancestry weighted by those contributions, i.e., minimize **c’θ c**, where **c** is a *N* × 1 vector of proportions of offspring left by each candidate (i.e., the vector of solutions), *N* is the number of candidates and **θ** is the coancestry matrix. A restriction was imposed in the optimization such as the sum of the contributions of males and females is the same and equal to ½, i.e., **Q’c** = ½ **1**, where **Q** is a (*N* × 2) known incidence matrix indicating the sex of the candidates with 0s and 1s, and 1 is a (2 × 1) vector of ones. The optimization problem was solved using Lagrangian multipliers [2,24]. Note that with this approach, **c** can contain negative values for some candidates. The contribution of candidates with *c_i_* < 0 was then set to 0 and the optimization was repeated with the remaining candidates until all elements of **c** were non-negative. Finally, the contribution of individual *i* (*c_i_*), which is a proportion, was converted to a number of offspring by multiplying *c_i_* by 2*N* and rounding to the nearest integer but ensuring that the number of offspring of each sex equals to *N*/2. Each parent was randomly allocated to different mates (among the selected individuals) to produce its offspring.

Two management strategies were investigated, and they differed in the genomic coancestry matrix used in the optimization of contributions. Under strategy S_O_LH_, the coancestry matrix used was matrix **θ_LH_** which describes the excess in the observed number of alleles shared by two individuals relative to the expected number under Hardy–Weinberg equilibrium [18,25]. Specifically, the coancestry coefficient between individuals *i* and *j* was computed as

(1) $$f_{LH\left(i,j\right)}=\frac{\sum_{k=1}^{S}f_{OBS\left(i,j\right)k}-S+2\sum_{k=1}^{S}p_{k}\left(1-p_{k}\right)}{2\sum_{k=1}^{S}p_{k}\left(1-p_{k}\right)}$$

where $f_{OBS\left(i,j\right)}$ is the proportion of alleles shared by individuals *i* and *j*, *S* is the number of SNPs and *p_k_* is the frequency of the reference allele (allele *B*) of SNP *k* in *t* = 0. Under strategy S_O_VR_, the coancestry matrix used was matrix **θ_VR_** which is based on the genomic relationship matrix obtained from VanRaden’s method 2 [19]. Specifically, the coancestry coefficient between individuals *i* and *j* was computed as

(2) $$f_{VR\left(i,j\right)}=\frac{1}{2S}\sum_{k=1}^{S}\frac{\left(x_{ki}-2p_{k}\right)\left(x_{kj}-2p_{k}\right)}{2p_{k}\left(1-p_{k}\right)}$$

where *x_ki_* is the genotype of individual *i* for SNP *k*, coded as 0, 1 or 2 for genotypes *AA*, *AB* and *BB*, respectively, and $p_{k}\;$is as defined for *f_LH_*.

In most scenarios, both coancestry matrices were computed every generation using all SNPs that were segregating in *t* = 0. However, we analyzed two additional scenarios where two different minor allele frequency (MAF) thresholds were imposed for the SNPs to be used to compute the coancestry matrices: (i) using only SNPs with MAF > 0.05; and (ii) using only SNPs with MAF > 0.25. The first threshold (MAF > 0.05) was considered because it is commonly applied when analyzing real data to reduce the number of potential genotyping errors. The second threshold (MAF > 0.25) was considered to explore the influence of rare alleles on the performance of the coancestry matrices investigated. It is known that with VanRaden’s method rare alleles contribute more to the coancestry coefficient than common alleles [21,26]. It is, thus, interesting to determine how the differences between management strategies S_O_LH_ and S_O_VR_ vary in the different MAF scenarios. Management in these additional scenarios was performed for 50 generations.

Furthermore, as a benchmark, we simulated a strategy (strategy *S_E_*) where the contributions of all candidates were equalized (i.e., all individuals contributed with two offspring to the next generation). This is the simplest management strategy that has been proposed to maintain genetic diversity by increasing *N_e_*. It should be noticed that when dealing with populations in which the relationships between individuals are homogeneous (all equally related), this strategy leads to a *N_e_* close to 2*N*.

### 2.3. Parameters Evaluated

Management strategies were compared in terms of the genetic variability retained and the trajectory of the allele frequencies across generations for the SNPs and for the unobserved loci. Moreover, strategies were compared in terms of the number of individuals selected to produce the next generation (*N_S_*) and the number of loci still segregating in a given generation, both for SNPs and for unobserved loci. The amount of genetic variability retained was measured as the expected heterozygosity (*H_e_*) computed as $1-\sum_{k=1}^{L}\sum_{l=1}^{2}p_{kl}^{2}$, where *L* is the number of loci (SNPs or unobserved loci) and $p_{lk}$ is the frequency of allele *l* of locus *k*.

In order to evaluate the ‘distance’ between frequencies in a given generation *t* and frequencies in *t* = 0, we used the Kullback–Leibler (*KL*) divergence criterion, which measures how different is a particular distribution from a reference distribution [27], which here is the distribution of allele frequencies in *t* = 0. The *KL* divergence between current frequencies and frequencies in *t* = 0 was computed as

(3) $$KL=\overset{L}{\underset{k=1}{\sum }}\overset{2}{\underset{l=1}{\sum }}{p^{\prime }}_{kl}log\frac{{p^{\prime }}_{kl}}{p_{kl}},$$

where $p_{kl}\;$is the frequency of allele *l* of locus *k* in *t* = 0, and ${p^{\prime }}_{kl}$ is the corresponding frequency in the current generation (*t* > 0). The summation over alleles included only alleles with ${p^{\prime }}_{kl}\;$> 0.

Finally, *N_e_* was estimated from the change in heterozygosity in SNP loci. Thus, *N_e_* in generation *t* was computed as *N_e_* = 1/2 Δ*H_e_*, where Δ*H_e_* equals $H_{e\left(t-1\right)}-H_{e\left(t\right)}/H_{e\left(t-1\right)}$. All results presented are averages over the 100 replicates.

## 3. Results

### 3.1. Expected Heterozygosity and Kullback–Leibler Divergence for Populations of Size N = 100

For populations of size *N* = 100, and using all the SNPs segregating in *t* = 0, strategy S_O_LH_ led to higher genetic variability (measured as *H_e_*) than strategy S_O_VR_ (Table 1) and the difference between both strategies increased across generations. In particular, *H_e_* was about 1%, 4% and 11% higher with S_O_LH_ than with S_O_VR_ in *t* = 1, 10 and 50, respectively. With S_O_LH_, *H_e_* even slightly increased in the initial generations while with S_O_VR_, *H_e_* decreased from the start. Moreover, *H_e_* obtained with strategy S_O_VR_ was very similar to *H_e_* obtained with strategy S_E_. Table 1 also shows that S_O_VR_ maintained allele frequencies closer to those in the base population than S_O_LH_ given that the *KL* values for S_O_LH_ were ≥ 100% higher than for S_O_VR_. The differences in *KL* between both strategies increased across generations. Moreover, at later generations, S_O_VR_ was slightly more efficient in maintaining the initial frequencies than S_E_, a strategy that is expected to maximize *N_e_* and, thus, to minimize genetic drift.

The use of both matrices (**θ_LH_** and **θ_VR_**) in OC also led to different numbers of individuals selected as parents of the next generation (*N_S_*). In particular, with S_O_LH_, between 10% and 30% fewer individuals were selected than with S_O_VR_ (Table 1). In fact, with the latter, almost all individuals were selected in all generations up to *t* = 10. The difference in *N_S_* entailed a difference in the number of loci that remained segregating across generations that was much higher with S_O_VR_ than with S_O_LH_ (Table 1), particularly in the initial generations. As for *H_e_* and for *KL*, strategies S_O_VR_ and S_E_ led to very similar values of *N_S_*. 

Table 2 shows the evolution across generations of the average frequency of the minor allele in *t* = 0. This average frequency was practically constant with S_E_ and slightly decreased with S_O_VR_. However, with S_O_LH_, it increased from ~1% in *t* = 1 to 16–19% in *t* = 50. Thus, it is clear that S_O_LH_ leads average frequencies upward (ultimately towards 0.5) and S_O_VR_ tends to maintain them. As expected, these patterns were more evident for the SNPs than for the unobserved loci.

**Figure 1:**
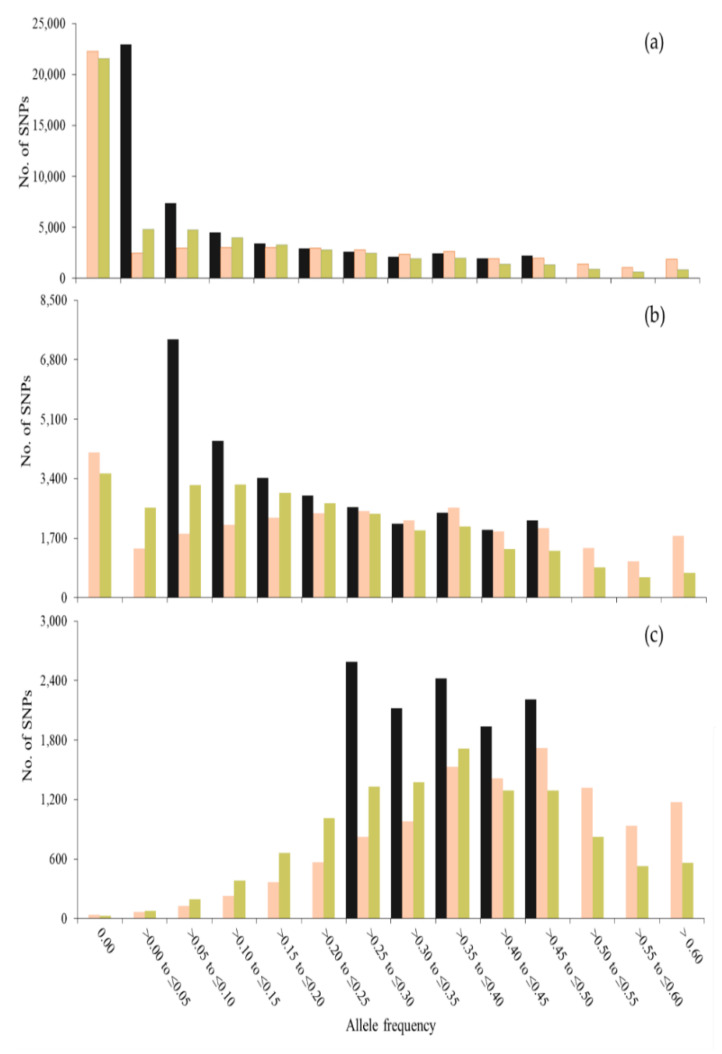
Number of SNPs for each class of allele frequency of the allele that was minor at generation 0 (gray bars) and the frequency of this allele after 50 generations, when contributions are optimized using Li and Horvitz (S_O_LH_, in orange) and VanRaden (S_O_VR_, in green) coancestry matrices computed with SNPs with MAF > 0.00 (**a**), MAF > 0.05 (**b**) and MAF > 0.25 (**c**) in a population of 100 individuals.

**Figure 2:**
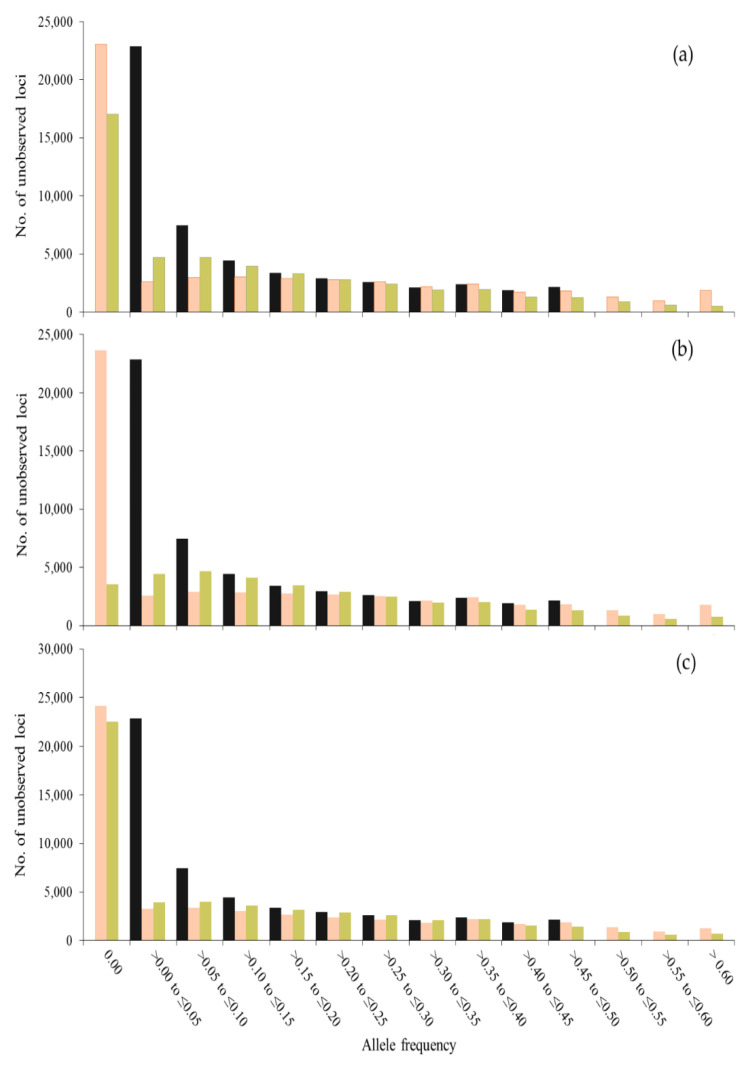
Number of unobserved loci for each class of allele frequency of the allele that was minor at generation 0 (gray bars) and the frequency of this allele after 50 generations, when contributions are optimized using Li and Horvitz (S_O_LH_, in orange) and VanRaden (S_O_VR_, in green) coancestry matrices computed with SNPs with MAF > 0.00 (**a**), MAF > 0.05 (**b**) and MAF > 0.25 (**c**) in a population of 100 individuals.

Figure 1 and Figure 2 show the frequency (*f*) distribution also for minor alleles in *t* = 0 in this generation and after 50 generations of management, using different sets of SNPs to compute coancestries. When using all SNPs segregating in *t* = 0, the distributions for SNPs and unobserved loci were very similar (Figure 1a and Figure 2a). However, when using only SNPs with MAF > 0.05 or MAF > 0.25, the distribution for SNPs was greatly affected. When using SNPs with MAF > 0 or MAF > 0.05 (Figure 1a,b), a greater number of SNPs was fixed with S_O_LH_ than with S_O_VR_ across generations (see class *f* = 0.00). However, more loci (SNPs and unobserved loci) with low frequencies (0.00 < *f* ≤ 0.15) were observed with S_O_VR_ than with S_O_LH_ and more loci with higher frequencies (*f* > 0.4) were observed with S_O_LH_ than with S_O_VR_. Thus, although more alleles are fixed with S_O_LH_, those that are kept segregating increase their frequency, while with S_O_VR_ the frequencies tend to be maintained. The highest difference between SNPs and unobserved loci was found when only SNPs with MAF > 0.25 were used to estimate the coancestry matrices (Figure 1c and Figure 2c). These differences are due to the fact that no MAF filtering was done for the unobserved loci.

**Figure 3:**
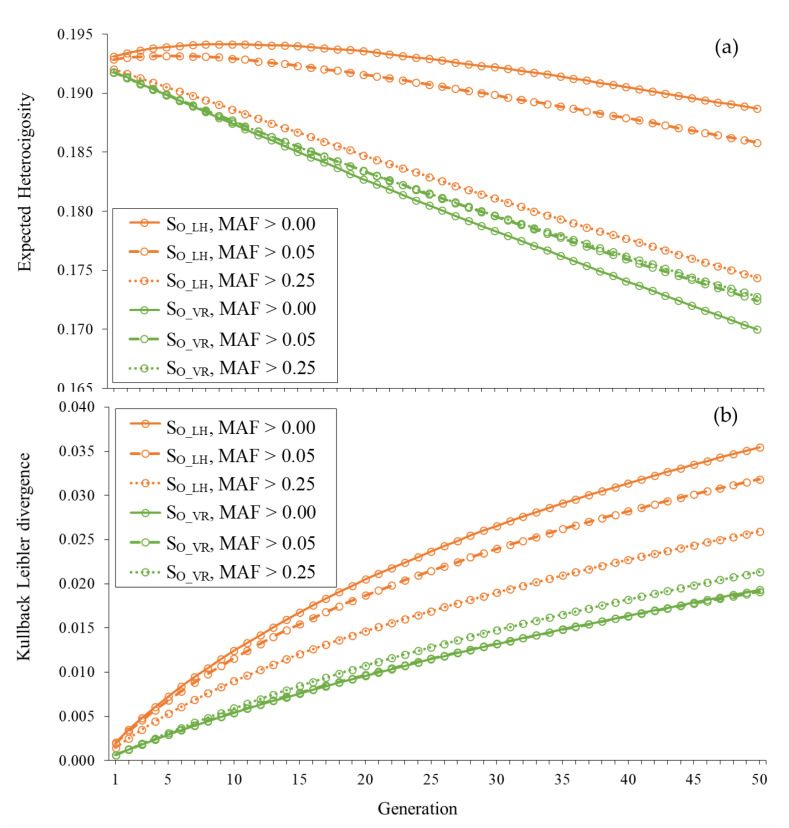
Expected heterozygosity (**a**) and Kullback–Leibler divergence (**b**) for unobserved loci across generations when contributions are optimized using Li and Horvitz (S_O_LH_) and VanRaden (S_O_VR_) coancestry matrices computed with SNPs with MAF > 0.00, MAF > 0.05 and MAF > 0.25 in a population of 100 individuals.

Figure 3 shows the trajectories of *H_e_* and *KL* across generations for unobserved loci under strategies S_O_LH_ and S_O_VR_ using the three different sets of SNPs. The heterozygosity maintained with S_O_LH_ decreased as the MAF criterion chosen for the SNPs used to estimate coancestries becomes more restrictive given that the number of SNPs used decreased. In fact, the small increase in *H_e_* observed in the initial generations when using all SNPs (MAF> 0.00) was not observed when using only the SNPs with MAF > 0.05 or MAF > 0.25. In parallel, the *KL* divergence with S_O_LH_ also decreased when increasing the severity of the restriction imposed on the SNPs used. However, with S_O_VR_, the changes observed in *H_e_* and *KL* when using a different set of SNPs were very small.

### 3.2. Expected Heterozygosity and Kullback–Leibler Divergence for Populations of Size N = 20 

Table 3 shows results from the different strategies (S_E_, S_O_LH_ and S_O_VR_) for populations of size *N* = 20, when all SNPs segregating in *t* = 0 were used in the management. Similar to the results found for populations of *N* = 100, (i) S_O_LH_ led to higher *H_e_* than S_O_VR_ and S_E_; and (ii) S_O_VR_ maintained allele frequencies closer to those in *t* = 0 than S_O_LH_. However, differences among strategies were smaller for populations of *N* = 20. For instance, for *N* = 20, *H_e_* in *t* = 10 was less than 1% higher when managing with S_O_LH_ than when managing with S_O_VR_, while for *N* = 100 this percentage was about 4%. For *KL*, the highest difference between strategies was 0.0027 units with *N* = 20 and 0.0127 units with *N* = 100. However, with *N* = 20, contrary to what happened with *N* = 100, S_O_LH_ managed to keep frequencies closer to the initial frequencies than S_E_ in the last generations (*t* ≥ 30).

In populations of size *N* = 20, individuals are more closely related than in populations of size *N* = 100 and the genetic variability is smaller. Thus, most (if not all) individuals were selected to be parents of the next generation with all management strategies across generations. It should be noted that the number of loci segregating in *t* = 0, when management started, was substantially smaller when simulating populations of size *N* = 20. In order to investigate if the differences observed between *N* = 20 and *N* = 100 are a consequence of the different number of loci segregating in *t* = 0, a scenario with *N* = 100 starting with the same number of SNPs as in the scenario with *N* = 20 (about 40,000 SNPs) was simulated. The results indicate that the differences between scenarios with different *N* were due to the population size and not to the different number of loci (results not shown).

### 3.3. Effective Population Size

Table 4 shows estimates of *N_e_* across generations for the different scenarios simulated. For *N* = 100, estimates of *N_e_* were around 200 individuals under strategies S_E_ and S_O_VR_. This is the expected value for *N_e_* when contributions are equalized since *N_e_* is approximately equal to 2*N*. However, under strategy S_O_LH_, estimates of *N_e_* were unreasonable as they took negative values in the initial generations. In later generations, *N_e_* became positive but did not reach a stable value. For *N* = 20, estimates under strategies S_E_ and S_O_VR_ were around 40 individuals, as expected. Estimates of *N_e_* under strategy S_O_LH_ were between 6% and 50% higher than under strategy S_E_.

## 4. Discussion

Using computer simulations, this study has compared two different management strategies in terms of two important criteria in genetic conservation programs, i.e., genetic diversity (*H_e_*) maintained and changes in allele frequencies. Both strategies optimize contributions for maintaining diversity but differ in the genomic coancestry matrix used in the optimization (**θ**_LH_ in strategy S_O_LH_ and **θ**_VR_ in strategy S_O_VR_). Moreover, as a benchmark, the simplest management strategy proposed to maintain genetic diversity that implies equalizing the contributions of all candidates (strategy S_E_) was evaluated.

The changes in allele frequencies were evaluated using the *KL* divergence criterion. The greater the value of *KL*, the greater the divergence of frequencies with respect to the frequencies in the base population. When the strategies were compared using the *KL* criterion, it was clear that strategy S_O_LH_ gives higher values than strategy S_O_VR_, indicating that the latter is able to maintain allele frequencies closer to the original frequencies (lower *KL* values). On the other hand, with strategy S_O_LH_, the population evolves differently as it pushes frequencies towards 0.5 and thus changes the genetic composition of the population more than strategy S_O_VR_.

Pushing frequencies towards 0.5 as strategy S_O_LH_ does leads to higher genetic variability when measured as expected heterozygosity. Thus, the hypothesis raised by Gómez-Romano et al. [21] that using matrix **θ**_LH_ in OC designed for maintaining genetic diversity better achieves the objective (i.e., higher *H_e_*) than using matrix **θ**_VR_, but using the latter maintains allele frequencies closer to the initial frequencies, is confirmed. This was observed both in populations with *N* = 20 and in populations with *N* = 100 although the differences between both strategies were smaller with *N* = 20. This is because individuals in the smaller populations are more closely related and there are less options to choose among individuals and strategies behave more similarly.

Saura et al. [9] showed that the use of the pedigree-based coancestry matrix in OC maintained allele frequencies close to those of the initial population. This is related to the high levels of *N_e_* obtained when minimizing pedigree coancestry (close to 2*N*), leading to reduced drift and little departures to the original frequencies. Additionally, several studies [10,12] have shown that OC based on pedigrees leads to less maintained genetic diversity than the use of genomic coefficients based on Nejati-Javaremi´s matrix [22]. This is due to the fact that genomic data provide realized estimates of coancestry, while pedigree data provide expected values. Therefore, results under the management of populations with OC using the pedigree-based coancestry matrix would be similar to those under S_O_VR_.

Strategy S_O_VR_ was only slightly more efficient for maintaining frequencies than strategy S_E_. This strategy tends to reduce the change in allele frequencies, which implies a reduced genetic drift [17]. The magnitude of drift is minimized when *N_e_* equals approximately 2*N*, and it is well known that, when managing the population using pedigree information (as said before), this is achieved by equalizing contributions [6,28]. The small advantage of S_O_VR_ in terms of maintaining frequencies over S_E_ arises from the fact that the former uses realized relationships and detects real differences between individuals while S_E_ assumes homogeneous relationships. Contrarily, S_O_LH_ does not minimize drift but maximizes *H_e_* by shifting frequencies towards 0.5. Thus, results from S_O_LH_ are quite different to those obtained under S_E_ in terms of the number of selected candidates and their optimal contributions.

Given that strategy S_O_LH_ brings the frequencies towards 0.5, *H_e_* increased in the initial generations and this led to negative estimates of *N_e_* in the largest population (*N* = 100). As generations go by, *N_e_* becomes positive but with unrealistic very high values without attaining an asymptotic value. This was also observed by Toro et al. [23] who questioned the meaning of *N_e_* when genomic coancestry matrices are used in OC. They showed an unpredictable behavior for *N_e_* when using the similarity genomic matrix of Nejati-Javaremi et al. [22], which has a correlation of 1 with the **θ**_LH_ matrix used here [5,16,29]. However, our results show that when using **θ**_VR_ in OC, estimates of *N_e_* were close to the expected value when equalizing contributions (approximately 2*N*). As has been discussed above, the results from strategy S_O_VR_ were very similar to those from strategy S_E_ given that both tend to minimize drift. For the smallest population considered (*N* = 20), estimates of *N_e_* were close to 2*N* not only with S_O_VR_ but also with S_O_LH_. In such a small population, there are fewer options to choose among individuals and most of them are selected to contribute (Table 3). Thus, the three strategies investigated led to similar results.

Strategy S_O_LH_ led to higher *H_e_* but also to a higher loss of segregating loci than strategy S_O_VR_. In the largest population (*N* = 100), the percentage of alleles lost for unobserved loci at *t* = 1 was 13% and 9% with S_O_LH_ and S_O_VR_, respectively (Table 1). The difference in both management strategies in terms of the number of alleles lost could be due to a different number of individuals selected to contribute to the next generation that was lower with S_O_LH_. It must be emphasized that the mean coancestry of each individual with all the candidates (including the individual), i.e., the marginal of the coancestry matrix, is a useful concept for understanding the different numbers selected with both strategies. This is because the marginal of the coancestry matrix is a measure of the ‘relevance’ of each individual, in terms of the degree of genetic information shared with the rest, and the optimal solutions will depend on all relationships between candidates. Its value is the same for all candidates when considering **θ**_VR_. Then, all candidates are equally useful and should be selected as it was observed minimizing the global coancestry through OC using **θ**_VR_ (strategy S_O_VR_). However, when considering **θ**_LH_, the average coancestry of individuals *AA* (homozygous for the minor allele) is lower than that of individuals *BB* (homozygous for the major allele), since individuals *AA* harbor genetic information that is underrepresented (i.e., they carry the rarer allele) and should be favored for selection and contributions. Therefore, OC using **θ**_LH_ minimize the objective function when selecting the same number of *AA* and *BB* candidates. This leads to an increase in the frequency of allele *A* (actually to 0.5 in a single generation in this example with only one locus) while frequencies stay unchanged when using **θ**_VR_.

Fernández et al. [13] claimed that OC management using coancestry matrices based on allele sharing moves frequencies to intermediate values and reduces the probability of losing alleles. In fact, these authors observed that strategies that maximize heterozygosity, by managing contributions from parents, keep levels of allelic diversity as high as strategies that maximize allelic diversity itself. Their results were obtained when applying OC using the similarity genomic matrix of Nejati-Javaremi et al. [22], calculated with up to 40 multiallelic markers, but the same could be expected when using **θ**_LH_ given that correlation between both matrices is 1. However, we have obtained solutions which maintain genetic diversity (*H_e_*) but result in a higher number of fixed loci and this could be due to the different numbers of markers used in both studies. 

To understand these contrasting results, we carried out extra simulations to compare observed with expected values for the number of fixed loci under both management strategies (i.e., S_O_LH_ and S_O_VR_). In this extra scenario, a population with *N* = 20 individuals was managed during four generations, with different numbers of SNPs used for the calculation of the coancestry matrices (20 and 1000). A single chromosome was simulated. The expected number of fixed SNPs (*ES_f_*) was estimated using the solutions that came out of each optimization before generating the offspring, following Fernández et al. [13]. Thus, *ES_f_* was computed as $\overset{2}{\underset{k=1}{\sum }}\overset{N}{\underset{i=1}{\prod }}prob_{ki}$, where $prob_{ki}$ is the probability of individual *i* not transmitting allele *k*. If parent *i* carries a unique type of allele (that is, homozygous for the *h* allele) and leaves descendants, $prob_{ki}$ is 0 if *k = h* and 1 if *k* *≠ h*. If it carries two different alleles (that is, heterozygous), the probability is $prob_{ki}={\left(0.5\right)}^{c_{i}}$, where *c_i_* is the number of offspring to be contributed by parent *i*. *ES_f_* value can be averaged then across loci. Table 5 shows that expected and observed numbers of SNPs becoming fixed each generation were close. When using only 20 SNPs, even though only seven–eight individuals are selected with S_O_LH_, the expected (observed) number of SNPs that become fixed is lower than with S_O_VR_. However, when the number of SNPs used was increased, the trend reversed and the expected (and observed) number of fixed SNPs becomes lower for S_O_VR_ than for S_O_LH_, even when the number of selected individuals increases for S_O_LH_. The explanation for this performance could be that, with many markers, S_O_LH_ is able to find a solution with higher mean *H_e_* by keeping loci with high MAF and allowing SNPs with rare alleles to become fixed. 

The results show that the differences in maintained diversity (*H_e_*) and divergence from the original frequencies (*KL*) between strategies S_O_LH_ and S_O_VR_ decreased when using only SNPs with a minimum MAF (MAF > 0.05 or MAF > 0.25) for computing the coancestry matrices. As mentioned above, S_O_LH_ promotes the contribution of individuals carrying rare alleles, as their coancestries with the rest of the population are smaller, and thus increases the frequencies of rare alleles. When the minimum MAF permitted increases, the number of rare alleles decreases, and the differences between the average coancestries between pairs of individuals decrease. In such situation, S_O_LH_ does not prioritize too much the contributions from any individual and leads to solutions that imply a higher number of candidates selected. Consequently, the results are closer to those obtained with strategy S_O_VR_. Moreover, when using only SNPs with high MAF in *t* = 0 (i.e., initial frequencies are close to 0.5), the performance of S_O_VR_ (i.e., keeping those initial frequencies) is similar to the performance of S_O_LH_ (moving them to intermediate values). These observations are in agreement with results from Morales-González et al. [16] and Villanueva et al. [29], who found that the correlation between VanRaden’s and Li and Horvitz’s coefficients increases with increasing the MAF of the SNPs used.

Here, we have optimized contributions of parents for minimizing the loss of variability and then changes in frequencies have been evaluated. On the other hand, Saura et al. [9] optimized contributions of parents for minimizing changes in allele frequencies and then the loss of genetic variability was evaluated. An alternative to both approaches could be to consider simultaneously the control of variability and the allele frequency changes. Similar to the OC algorithm designed for maximizing genetic gain while restricting the rate of inbreeding [2,3,24] or for maximizing the phenotypic level for a trait of interest while restricting the loss in variability when creating base populations [30], one could develop an algorithm for minimizing the loss of variability while restricting the change in frequencies or, alternatively, for minimizing frequency changes while restricting the loss of variability. The specific objective would depend on the particular interest of the managers of the program. This kind of approach was followed by Fernández et al. [31] in the context of optimizing the sampling strategy for establishing a gene bank. In particular, they developed an algorithm that simultaneously allows targeting frequencies for alleles at a particular locus while controlling the genetic diversity of other unlinked loci.

It could be also possible to combine both coancestry matrices (**θ**_LH_ and **θ**_VR_) in the objective function when the specific objective differs across genomic regions (i.e., in some regions the interest may be to maintain diversity, and in other regions the interest may be to maintain frequencies). Maintaining diversity may be of interest for regions associated with inbreeding depression for fitness-related traits and also for regions that harbor loci involved in general resistance to diseases (e.g., the major histocompatibility complex, MHC) as a high level of genetic diversity is desirable to ensure that the population can deal with potential new disease challenges [21]. Maintaining frequencies may be of interest in regions containing loci that have been under natural or artificial selection, and one wants to keep the genetic progress obtained. Gómez-Romano et al. [21] showed that the OC method using a matrix equivalent to **θ**_LH_ is efficient in maintaining *H_e_* in specific regions and simultaneously restricts the loss of *H_e_* in the rest of the genome. Their approach could be extended to include the use of **θ**_VR_ for minimizing the change in allele frequencies in some genomic regions. However, it has to be kept in mind that the higher the number of different parameters to be controlled, or the more regions to be treated differently, the lower the control of each objective one can expect.

In a conservation program, the maintenance of genetic variability throughout the genome is the general aim because usually there is no information available on the relevance of each genome region and the current or future use of the genetic variability present in particular regions. Therefore, it is better to conserve as much diversity as possible because if alleles are lost in a population, they will be no longer available. However, this strategy can lead to the maintenance or even an increase in the frequency of deleterious alleles. Different methods have been proposed to avoid this when using the OC method, including (i) selection of the best sib from the group of offspring generated by the selected parents [28] and (ii) combining selection with inbred matings [14] to allow for some kind of purging. Sonesson et al. [32] also proposed a model in which they tried to eliminate a disease from a population in different scenarios by explicitly performing selection against this condition. Currently, genomics can provide information on deleterious variability and the loci determining the occurrence of the disease [33], so a strategy where selection is made against these deleterious alleles [17], while you restrict the loss of variability in the rest of the genome, could be possible.

The amount of genetic variability retained was measured as the expected heterozygosity (*H_e_*). However, other measures such as allelic diversity can be used [13,34]. Allelic diversity is essential from an evolutionary perspective, since the limit of selection response is determined by the initial number of alleles [35,36]. It is worth noting that strategy S_O_VR_ would be more efficient than strategy S_O_LH_, not only to maintain allele frequency but also to maintain diversity when this is measured as the number of unobserved loci segregating. It is thus clear that the coancestry matrix to be used in OC when managing a particular genetic conservation program would be case specific.

Finally, it is worth mentioning that further work is needed to explore how the relaxation of some of the assumptions implicit in our simulations could affect the results obtained. Extra work would be necessary to investigate schemes with overlapping generations, variable population size over the management time frame, and different degrees of relatedness between the founders.

## 5. Conclusions

When applying strategy S_O_LH_, more *H_e_* is maintained than when applying strategy S_O_VR_ given that S_O_LH_ moves allele frequencies towards 0.5. However, S_O_VR_ maintained allele frequencies closer to those of the initial generation and more loci segregating than S_O_LH_. Therefore, considering that conservation programs generally aim to increase genetic diversity, but it is also important to maintain population uniqueness, the choice of which genomic coancestry matrix is used in management may depend on which of these two goals is more important for each particular case. When a subset of SNPs with MAF > 0.05 or MAF > 0.25 is used to estimate coancestry matrices, the differences between both strategies in terms of both *H_e_* and *KL* were reduced. The differences between strategies were smaller for populations of smaller sizes given that in a smaller population it is more difficult to differentiate between individuals.

## Figures and Tables

**Table 1:** Expected heterozygosity (*H_e_*, in %) and Kullback–Leibler divergence for unobserved loci (*KL* × 10^2^), number of selected candidates (*N_S_*), and number of SNPs (*S*) and unobserved loci (*U*) segregating across generations (*t*) when contributions are equalized (S_E_) and when they are optimized using Li and Horvitz (S_O_LH_) and VanRaden (S_O_VR_) coancestry matrices computed with SNPs with MAF > 0.00 in a population of 100 individuals.

	S_E_						S_O_LH_ *						S_O_VR_ *				
*t*	*H_e_*	*KL*	*N_S_*	*S*	*U*		*H_e_*	*KL*	*N_S_*	*S*	*U*		*H_e_*	*KL*	*N_S_*	*S*	*U*
1	19.17	0.06	100	51,035	50,894		+0.14	+0.14	−39	−2239	−2246		0.00	0.00	0	+8	+18
2	19.12	0.12	100	49,873	49,737		+0.21	+0.23	−36	−3206	−3229		0.00	0.00	0	−22	0
3	19.07	0.18	100	48,852	48,729		+0.28	+0.30	−35	−3792	−3847		0.00	0.00	0	−61	−52
4	19.03	0.24	100	47,946	47,828		+0.35	+0.37	−35	−4182	−4261		0.00	0.00	−1	−113	−101
5	18.98	0.30	100	47,108	47,003		+0.41	+0.43	−33	−4384	−4499		0.00	−0.01	−1	−162	−157
10	18.73	0.57	100	43,777	43,691		+0.68	+0.68	−30	−4731	−4975		0.00	−0.03	−2	−399	−401
15	18.51	0.82	100	41,311	41,217		+0.89	+0.86	−28	−4523	−4855		−0.01	−0.06	−5	−595	−587
20	18.27	1.06	100	39,313	39,229		+1.08	+0.99	−26	−4152	−4567		−0.01	−0.09	−6	−714	−720
30	17.82	1.50	100	36,231	36,140		+1.40	+1.16	−24	−3329	−3896		+0.01	−0.18	−9	−906	−899
40	17.38	1.90	100	33,854	33,759		+1.67	+1.24	−22	−2517	−3215		+0.03	−0.26	−11	−995	−970
50	16.95	2.28	100	31,940	31,848		+1.92	+1.27	−21	−1786	−2594		+0.05	−0.35	−12	−1081	−1036

* S_O_LH_ and S_O_VR_ values are those deviated from S_E_. Standard errors (computed across replicates) ranged from 4.91 × 10^−5^ to 9.54 × 10^−5^ for *H_e_* and from 0.16 × 10^−5^ to 7.39 × 10^−5^ for *KL*.

**Table 2:** Average frequency of the minor allele in generation 0 (× 10^2^) across generations (*t*) for SNPs and unobserved loci when contributions are equalized (S_E_) and when they are optimized using Li and Horvitz (S_O_LH_) and VanRaden (S_O_VR_) coancestry matrices in a population of 100 individuals.

		SNPs				Unobserved Loci		
*t*		S_E_	S_O_LH_	S_O_VR_		S_E_	S_O_LH_	S_O_VR_
0		13.45	13.45	13.45		13.39	13.39	13.39
1		13.44	13.68	13.45		13.39	13.60	13.40
2		13.44	13.81	13.45		13.39	13.72	13.40
3		13.44	13.94	13.45		13.38	13.82	13.39
4		13.44	14.06	13.44		13.38	13.93	13.39
5		13.44	14.17	13.44		13.38	14.02	13.39
10		13.44	14.67	13.41		13.38	14.44	13.36
15		13.45	15.08	13.37		13.39	14.77	13.33
20		13.44	15.42	13.32		13.39	15.05	13.29
30		13.44	15.96	13.23		13.39	15.46	13.23
40		13.45	16.36	13.12		13.39	15.75	13.15
50		13.45	16.67	13.01		13.40	15.98	13.07

**Table 3:** Expected heterozygosity (*H_e_*, in %) and Kullback–Leibler divergence for unobserved loci (*KL* × 10^2^), number of selected candidates (*N_S_*), and number of SNPs (*S*) and unobserved loci (*U*) segregating across generations (*t*) when contributions are equalized (S_E_) and when they are optimized using Li and Horvitz (S_O_LH_) and VanRaden (S_O_VR_) coancestry matrices computed with SNPs with MAF > 0.00 in a population of 20 individuals.

	S_E_						S_O_LH_ *						S_O_VR_ *				
*t*	*H_e_*	*KL*	*N_S_*	*S*	*U*		*H_e_*	*KL*	*N_S_*	*S*	*U*		*H_e_*	*KL*	*N_S_*	*S*	*U*
1	23.35	0.27	20	38,995	38,955		+0.04	+0.05	−1	−193	−233		+0.03	0.00	0	+31	+134
2	23.06	0.52	20	37,093	37,050		+0.06	+0.07	−1	−275	−335		+0.01	0.00	0	+52	+155
3	22.76	0.76	20	35,522	35,472		+0.10	+0.09	−1	−356	−410		−0.02	+0.01	0	−12	+104
4	22.48	0.99	20	34,166	34,119		+0.07	+0.11	−1	−390	−442		−0.02	−0.01	0	−16	+94
5	22.19	1.20	20	33,016	32,978		+0.08	+0.13	−1	−456	−528		−0.03	0.00	0	−69	+37
10	20.79	2.17	20	28,782	28,692		+0.17	+0.18	−1	−533	−563		−0.07	−0.03	−1	−269	−62
15	19.52	3.00	20	25,844	25,763		+0.24	+0.17	−1	−497	−563		−0.03	−0.07	−1	−400	−206
20	18.33	3.75	20	23,512	23,434		+0.37	+0.13	−1	−336	−424		−0.01	−0.12	−1	−429	−247
30	16.02	5.13	20	19,854	19,795		+0.79	−0.02	−2	+81	−59		+0.04	−0.25	−2	−469	−337
40	14.03	6.26	20	17,044	17,002		+1.15	−0.16	−1	+545	+377		+0.18	−0.43	−2	−432	−309
50	12.32	7.23	20	14,853	14,811		+1.39	−0.27	−1	+787	+592		+0.19	−0.52	−2	−433	−322

* S_O_LH_ and S_O_VR_ values are those deviated from S_E_. Standard errors (computed across replicates) ranged from 1.15 × 10^−4^ to 3.37 × 10^−4^ for *H_e_* and from 10 × 10^−4^ to 1.72 × 10^−4^ for *KL*.

**Table 4:** Effective population size (*N_e_*) across generations (*t*) when contributions are equalized (S_E_) and when they are optimized using Li and Horvitz (S_O_LH_) and VanRaden (S_O_VR_) coancestry matrices in populations of different sizes (*N*).

		*N* = 100			*N* = 20		
*t*		S_E_	S_O_LH_	S_O_VR_	S_E_	S_O_LH_	S_O_VR_
1		188.21	−111.90	195.55	36.92	42.27	40.40
5		199.07	−855.78	197.46	36.78	41.24	34.31
10		191.56	−5777.32	193.05	38.54	40.81	41.77
15		203.50	1855.71	194.54	36.65	45.41	43.18
20		202.62	1033.03	201.52	40.61	47.25	40.02
25		190.44	636.00	209.85	40.20	47.08	42.02
30		193.58	670.07	209.79	36.45	53.03	38.57
35		193.30	524.97	206.03	33.41	50.28	44.62
40		204.95	601.67	212.53	36.94	47.91	49.68
45		207.44	703.31	205.00	37.52	48.50	40.09
50		206.86	481.08	213.02	41.99	46.20	38.53

**Table 5:** Number of selected candidates (*N_S_*) and expected (*ES_f_*) and observed number of fixed SNPs (*S_f_*) across generations (*t*) when contributions are optimized using Li and Horvitz’s (S_O_LH_) and VanRaden’s (S_O_VR_) coancestry matrices computed with two different number of SNPs (*S*), for a population of 20 individuals.

		S_O_LH_				S_O_VR_		
*t*	*S*	*N_S_*	*ES_f_*	*S_f_*		*N_S_*	*ES_f_*	*S_f_*
1	20	7	0.3	0		20	0.3	0
2		7	0.7	0		13	0.8	1
3		8	0.8	0		13	1.4	1
4		8	0.9	0		12	1.7	1
1	1000	15	21.7	21		20	17.6	18
2		16	38.9	37		19	34.6	33
3		15	54.6	52		19	50.9	47
4		15	68.6	64		18	66.3	60

## Data Availability

The codes to perform the simulations are available from the corresponding author on reasonable request.
